# Supplementary material for: Bayesian refinement of protein structures and ensembles against SAXS data using molecular dynamics
Source: PLoS Comput Biol. 2017 Oct 18;13(10):e1005800. doi: 10.1371/journal.pcbi.1005800 (PMC5662244; doi:10.1371/journal.pcbi.1005800)
Supplement: S1 Archive — (BZ2) [file pcbi.1005800.s011.tar.bz2 › gromacs_4.62_bayesian_saxs_refi/share/html/online/gmxdemo.html]

Getting started - Gmxdemo


| Getting started - Gmxdemo Main Table of Contents |

**VERSION 4.6  
Sat 19 Jan 2013**

---

## The GROMACS demo

The demo is designed to demonstrate the user-friendlyness
of the GROMACS software package. The only non-friendly part is
that it requires the C shell to run the script. If your shell
is e.g. bash (common on Linux), first start the C shell with the
command 'tcsh'. To run the demo, first move
to your tutor/gmxdemo directory:
  
  

|  |  |  |
| --- | --- | --- |
|  | cd tutor/gmxdemo   \| |

  
And then start the demo script:
  
  

|  |  |  |
| --- | --- | --- |
|  | demo   \| |

  
This demo handles a complete Molecular Dynamics simulation of a
peptide in water, starting from a pdb
structure. You will be asked to press return a few times, and to run the
trajectory viewer. Please read the instructions as you go along.

### More Info

More info can be found in the
flowchart
(for a quick overview) and the
GROMACS FAQ (Frequently asked questions).

  

---

  


### Go to the next step


---
